# Nutrient requirements and evaluation of equations to predict chemical body composition of dairy crossbred steers

**DOI:** 10.5713/ajas.19.0829

**Published:** 2020-06-24

**Authors:** Flávia Adriane de Sales Silva, Sebastião de Campos Valadares Filho, Luiz Fernando Costa e Silva, Jaqueline Gonçalves Fernandes, Bruno Corrêa Lage, Mario Luiz Chizzotti, Tara Louise Felix

**Affiliations:** 1 Department of Animal Science, Universidade Federal de Viçosa, Viçosa 36570-900, Brazil; 2 Alltech do Brasil Agroindustrial, Maringá 87050220, Brazil; 3 Department of Statistics, Universidade Federal de Lavras, Lavras 37200-000, Brazil; 4 Department of Animal Science, Pennsylvania State University, University Park, State College, PA 16802, USA

**Keywords:** Carcass Composition, Cattle, Body Composition

## Abstract

**Objective:**

Objectives were to estimate energy and protein requirements of dairy crossbred steers, as well as to evaluate equations previously described in the literature (HH46 and CS16) to predict the carcass and empty body chemical composition of crossbred dairy cattle.

**Methods:**

Thirty-three Holstein×Zebu steers, aged 19±1 months old, with an initial shrunk body weight (BW) of 324±7.7 kg, were randomly divided into three groups: reference group (n = 5), maintenance level (1.17% BW; n = 4), and the remaining 24 steers were randomly allocated to 1 of 4 treatments. Treatments were: intake restricted to 85% of *ad libitum* feed intake for either 0, 28, 42, or 84 d of an 84-d finishing period.

**Results:**

The net energy and the metabolizable protein requirements for maintenance were 0.083 Mcal/EBW^0.75^/d and 4.40 g/EBW^0.75^, respectively. The net energy (NE_G_) and protein (NP_G_) requirements for growth can be estimated with the following equations: NE_G_ (Mcal/kg EBG) = 0.2973_(±0.1212)_ ×*EBW*^0.4336(±0.1002)^ and NP_G_ (g/d) = 183.6_(±22.5333)_×EBG−2.0693_(±4.7254)_×RE, where EBW, empty BW; EBG, empty body gain; and RE, retained energy. Crude protein (CP) and ether extract (EE) chemical contents in carcass, and all the chemical components in the empty body were precisely and accurately estimated by CS16 equations. However, water content in carcass was better predicted by HH46 equation.

**Conclusion:**

The equations proposed in this study can be used for estimating the energy and protein requirements of crossbred dairy steers. The CS16 equations were the best estimator for CP and EE chemical contents in carcass, and all chemical components in the empty body of crossbred dairy steers, whereas water in carcass was better estimated using the HH46 equations.

## INTRODUCTION

It is recognized that dairy (Holstein and Holstein crossbreeds) and beef cattle exhibit differences regarding their nutritional requirements [1,2]. However, despite the large participation of male dairy cattle in feedlots in US and Brazil [3–5], most of nutritionists in US (92.2%) and Brazil (71.7%) use some version beef cattle NRC as source of information on nutrient requirements to formulate diets [3,6]. Therefore, the establishment of proper nutritional requirements for male dairy cattle and its crossbreeds is necessary and consequently ensures their subsequent productive performance and farmers profitability.

In this regard, quantifying body composition is a major obstacle when estimating nutrient requirements. The direct determination of body composition, which is based on the separation and dissection of all body constituents, and analysis of its individual constituents, is the most accurate method available, to generate reliable data. However, this process is lengthy, laborious, and expensive. Due to these challenges, indirect methods have been developed for estimating body composition [7–9].

Among the indirect methods available, the method that predicts body composition from equations based on the 9th to 11th rib section composition, which was first proposed by Hankins and Howe (HH46) [7], is wide spread due the ease of use, reduced cost involved, and speed of the process. However, Paulino et al [10] and Marcondes et al [11] unanimously concluded that the equations developed by HH46 [7] were not fully applicable to predict body composition of Zebu cattle. Prediction equations to estimate the growth pattern and composition of gain of Holstein×Zebu crossbreds have not been reported. In this respect, Costa e Silva et al (CS16) [2] developed equations for estimating body composition of crossbred dairy cattle. Nonetheless, they still need to be evaluated with an independent database in order to be widely and reliably adopted. Therefore, testing those equations for crossbred dairy animals should provide useful information about their utility for such animals. Thus, the objectives were to estimate energy and protein requirements of dairy crossbred steers, as well as to evaluate the goodness of fit of prediction equations developed by HH46 [7] and CS16 [12] to predict the carcass and empty body chemical composition of crossbred dairy cattle.

## MATERIALS AND METHODS

The animal care and use committee of the Universidade Federal de Viçosa approved all procedures involving animals (protocol number 12/2016).

### Location, animals resource, study design, diet, and feed composition

Thirty-three ¾ Holstein×Zebu steers (average age = 19±1 months; average initial body weight [BW] = 324±7.7 kg) were used in this study. Gyr was the Zebu breed utilized in the crossbreeding. Five steers were assigned to a baseline group (BW = 341±21.1), 4 were fed at maintenance (1.17% BW; BW = 328±23.6), whereas 24 steers (BW = 320±8.3) were randomly allotted to 1 of 4 treatments (n = 6 steers per treatment). Treatments were: intake restricted at 85% of *ad libitum* dry matter (DM) for 0 (R0) or no restriction, 28 (R28), 42 (R42), or 84 (R84) d of an 84-d finishing period. These strategies were needed to obtain sufficient variation in the body composition to mimic animal growth patterns.

The steers were housed in 6 m^2^ individual pens with a concrete floor, which were equipped with individual feeders and continuous flow concrete drinkers. The experimental diet was the same for all treatments. The diet was calculated according to BR CORTE recommendations [2] to provide approximately 11% crude protein (CP) on a DM basis and to support an average daily gain (ADG) of 1.4 kg. The diet contained (DM basis) 40% corn silage and 60% of a concentrate mix (Table 1).

The feed was provided once a day (0800 h) as total mixed ration, and the steers had free access to clean water. The diet for R0 steers was adjusted daily to provide orts of approximately 2% (as fed basis) of the total offered, and orts were mixed with the new feed that was offered in the next day. Briefly, bunks were evaluated at 0700 h daily to quantify refusals. According to the amount of refusals, the total mixed ration was reduced (more than 2% orts at morning evaluation) or increased (less than 2% orts at morning evaluation) to reach *ad libitum* intake. Silage was sampled daily and stored at −20°C until further analysis. Silage samples were combined weekly (percent as-fed basis), dried in a forced-air oven (55°C) for 72 h, and ground through a 1-mm screen (Fortinox, Piracicaba, SP, Brazil). The individual ingredients used to prepare the concentrate were sampled directly from the feed mill silos on the days the concentrate was mixed. These ingredients were analyzed individually and used to calculate the diet composition.

### Digestibility trials, slaughter and sampling procedures

To evaluate apparent total-tract digestibility and consequently the total digestible nutrients (TDN), two digestibility trials were performed, from d 25 to 27 and d 73 to 75, by collecting the total feces excreted over three consecutive days [13]. In brief, the feces were collected using dustpans and then stored in plastic buckets at room temperature. At the end of each collection day, feces were quantified and mixed. Sub-samples of approximately 300 g of feces (on an as-is basis) were saved each day. The fecal samples were dried in a forced-air oven (55°C) for 72 h, and ground through a 1-mm screen (Fortinox, Brazil). Furthermore, each period, an individual composite fecal sample from the 3 d of collection was prepared for each animal based on the DM content of the feces collected on the individual days. The final composite samples represented the 3-d collection of feces on a proportional DM basis.

All animals were slaughtered after 84 d. Steers were fasted for 14 h before slaughter. The slaughter was performed by stunning using the captive bolt technique followed by jugular vein exsanguination. The digestive tract of each steer was emptied and washed, and each organ was weighed separately. The weight of the heart, lungs, liver, spleen, kidneys, internal fat (fat surrounding kidneys, pelvis, and heart), diaphragm, mesentery, tail, trachea, esophagus, and reproductive tract along the weight of the washed gastrointestinal tract were added to that of the other body parts (head, hide, limbs, and blood) to determine the non-carcass components weight. The non-carcass weight was added to that of the carcass to determine the empty body weight (EBW). Blood samples were collected immediately after slaughter. Samples of blood, organs and viscera, head and limbs, and hide were placed in aluminum trays and freeze-dried (Liotop, LP 510 model, São Carlos, SP, Brazil).

The carcass of each steer was split in to 2 halves along the spine, and then cooled at −4°C for 24 h. After 24 h, the carcass halves were weighed. The 9th to 11th rib section was obtained from the left half, as recommended by HH46 [7], and subsequently dissected into bone, fat and muscle tissues. The rest of the left carcass was completely dissected into bone, muscle, and fat tissues, and the 9th to 11th rib section was summed, to obtain the full carcass composition. Dissected samples were weighed, individually ground, packed in aluminum trays, and freeze-dried (Liotop, LP 510 model, Brazil).

All lyophilized samples from the steer’s body were ground in a Wiley mill (Fortinox, Brazil) using liquid N. Four sub-samples per steer, named “carcass”, “non-carcass”, “muscle plus fat-rib section”, and “bones-rib section” were taken for further determination of the chemical composition. The carcass sample was comprised of the lyophilized samples of bone and muscle plus fat from the carcass. The non-carcass sample was comprised of blood, head and limbs, organs and viscera, and hide. The muscle plus fat-rib section was comprised of muscle and fat from the 9th to 11th rib section, and the bones-rib section contained the bone sample from the 9th to 11th rib section.

### Laboratory analysis and calculations

Individual feed ingredients and feces were analyzed for DM, organic matter (OM), CP, and ether extract (EE) according to the AOAC [14,15]. The quantification of neutral detergent fiber (NDF) was performed without the addition of sodium sulfite, but with the addition of thermostable α-amylase to neutral detergent (Ankom Tech. Corp., Fairport, NY, USA). The NDF concentrations were corrected for ash [16] and residual N compounds [17]. The non-fiber carbohydrates were calculated as proposed by Detmann and Valadares Filho [18]. The TDN of the diet was estimated by the sum of digestible nutrients as proposed by NRC [19].

Digestible energy (DE) intake was obtained by multiplying digestible nutrients by their respective energy values [19]. The metabolizable energy intake (MEI) was estimated as 82% DE [20]. The metabolizable protein intake (MPI) was obtained by adding the true digestible microbial CP (TDMCP) and the digestible RUP (DRUP) intake. The microbial CP (MCP) was calculated as proposed by Valadares Filho et al [2]. In addition, we assumed that the MCP is composed of 80% of AA and that these AA have an 80% digestibility [19]. The DRUP intake was estimated based on the difference between CP intake and MCP and multiplied by its intestinal digestibility (80%) [19].

Carcass, non-carcass, muscle plus fat-rib section, and bones-rib section samples were analyzed for DM [14], and CP and EE content [21]. The relationship between the shrunk BW (SBW) and EBW was calculated to convert SBW to EBW, while the relationship between the ADG and empty body gain (EBG) was calculated to convert ADG to EBG.

Whole-body energy content (EC) of each steer was obtained from the whole-body content for CP and fat by using their caloric equivalent [22]. The whole-body retained energy (RE) was calculated as the difference between the final and initial EC of each steer. Heat production (HP) was estimated as the difference between MEI and EC. Then, the net energy requirement for maintenance (NE_M_) was estimated to be the intercept (β_0_) of the exponential non-linear regression between HP and MEI [4]:

$$\text{HP }(\text{Mcal}/{\text{EBW}}^{0.75}/\text{d})=\beta 0\times {\text{e}}^{\left(\beta 1\times \text{MEI}\right)},$$

where HP (Mcal/EBW^0.75^/d), MEI (Mcal/EBW^0.75^/d), β0 and β1 = regression parameters; and e = the Euler number (2.718281). Also, the ME requirement for maintenance (ME_M_, Mcal/EBW^0.75^/d) was estimated by the iterative method, with ME_M_ considered to be the value where MEI equals HP [2]. The efficiency of utilization of ME_M_ (k_M_) was calculated as the ratio between the NE_M_ and ME_M_ [2].

The EC as a function of EBW of the animals, was estimated according to the exponential model [22]:

$$\text{EC}=\beta 0\times {\text{EBW}}^{\beta 1}.$$

The net energy requirement for gain (NE_G_) was obtained by the derivative of the equation above [22]:

$${\text{NE}}_{\text{G}}=\beta 0\times \beta 1\times {\text{EBW}}^{\left(\beta 1-1\right)},$$

where NE_G_ (Mcal/kg of EBG), EBW (kg). The efficiency of utilization of ME_G_ (k_G_) was estimated to be the slope (β1) of the linear regression between RE and MEI:

$$\text{RE }(\text{Mcal}/{\text{EBW}}^{0.75}/\text{d})=\beta 1\times \text{MEI}+\beta 0,$$

where RE (Mcal/EBW^0.75^/d), MEI (Mcal/EBW^0.75^/d). The metabolizable energy requirement for gain (ME_G_, Mcal/EBW^0.75^/d) was calculated by dividing the NE_G_ by k_G_ [2].

The metabolizable protein requirement for maintenance (MP_M_) was estimated to be the intercept (β0) of the linear regression between MPI and EBG divided by average metabolic EBW (EBW^0.75^) [2], as follows:

MPI=β1×EBG+β0,

where MPI = metabolizable protein intake (g/d), EBG (kg/d), and β0 and β1 = regression parameters.

To calculate the net protein requirement (NP_G_), we adjusted the model:

$${\text{NP}}_{\text{G}}=\beta 1\times \text{EBG}+\beta 2\times \text{RE},$$

where NP_G_ = net protein requirement for gain (g/d), EBG = empty body gain (kg/d), RE = retained energy (Mcal/d), and β1 and β2 = regression parameters. The efficiency of metabolizable protein utilization for gain (k_PG_) was estimated to be the slope (β1) of the equation:

RP=β1×MPI+β0,

where RP = whole-body retained protein (g/EBW^0.75^/d), MPI = metabolizable protein intake (g/EBW^0.75^/d), and β0 and β1 = regression parameters.

### Evaluated equations

The descriptive statistics of the variables used to estimate the carcass and empty body chemical composition are presented in Table 2. The equations developed CS16 [12] were used to estimate carcass and empty body chemical composition using the 9th to 11th rib section (Table 3). The equations developed HH46 [7] were used to estimate carcass chemical composition using the 9th to 11th rib section only. It is important to highlight that the present data was independent of those used to develop the predictive equations from HH46 [7] and CS16 [12].

### Statistical analysis

The relationship between the SBW and EBW and the ADG and EBG, and data on energy and protein requirements were analyzed as linear and nonlinear models built through the REG and NLIN procedures of SAS (version 9.4, SAS Inst. Inc.; Cary, NC, USA), respectively, in which the nonlinear model was adjusted with the Gauss-Newton method.

The body components estimated by HH46 [7] and CS16 [12] equations were compared with the observed values using the following regression model: y = β_0_+β_1_×x, where x = predicted values; y = observed values; β_0_ and β_1_ = intercept and slope, respectively. The regression was evaluated according to the following statistical hypothesis: H_0_: β_0_ = 0 and H_0_: β_1_ = 1, and H_a_: not H_0_. If the null hypotheses were not rejected, it could be concluded that the equations accurately and precisely estimate the body components of Holstein×Zebu steers. Slope and intercept were separately evaluated to observe possible errors in the equations.

Estimates were also evaluated using the mean square error of prediction (MSEP) and its components [23]:

$$\begin{array}{l} \text{MSEP}=\text{SB}+\text{MaF}+\text{MoF}=1/n\sum_{i=1}{\left(x_{i}-y_{i}\right)}^{2};\\ \text{SB}={\left(x_{i}-y_{i}\right)}^{2};\\ \text{MaF}={\left(s_{x}-s_{y}\right)}^{2};\\ \text{Mof}=2s_{x}s_{y}(1-r); \end{array}$$

where *x* are predicted values; *y* are observed values; MSEP is the mean squared error of prediction; SB is the squared bias; MoF is the component relative to the model of random fluctuation; MaF is the component relative to the magnitude of random fluctuation; *s_x_* and *s_y_* are the standard deviations of the predicted and observed values, respectively, and *r* is the Pearson linear correlation coefficient between the predicted and observed values. The prediction of efficiency was determined by estimating the correlation and concordance coefficient or reproducibility index (CCC) as described by Tedeschi [24]. Values of CCC below 0.4, between 0.4 and 0.7, and above 0.7 were considered low, medium, and high, respectively. All calculations were obtained using the Model Evaluation System (MES) [24]. For comparisons, the level of 0.05 was established as the critical level of probability for type I error.

## RESULTS AND DISCUSSION

### Energy and protein requirements

The average ratio between EBW and SBW and EBG and ADG observed in this experiment were 0.88 and 0.93, respectively (R^2^ of 0.98 and 0.97, respectively). The relationship between HP and MEI was described by the equation:

$$\text{HP}=0.083\times e^{\left(3.2917_{\left(\pm 0.1222\right)}\times MEI\right)}.$$

Thus, the NE_M_ was 0.083 Mcal/EBW^0.75^/d, while ME_M_ was 0.125 Mcal/EBW^0.75^/d for Holstein×Zebu steers. Therefore, the k_M_ was 0.66. The NE_G_ can be estimated with the equation:

$${\text{NE}}_{\text{G}}\;(\text{Mcal}/\text{kg EBG})=0.2973_{\left(\pm 0.1212\right)}\times {EBW}^{0.4336_{\left(\pm 0.1002\right)}}.$$

The k_G_ was 0.35, and this value was obtained based on the slope of the linear relationship between RE and MEI.

The MP_M_ was 4.40 g/EBW^0.75^, and this value was obtained based on a linear regression between MPI and EBG divided by the average metabolic EBW. The NP_G_ were estimated from the model:

$${\text{NP}}_{\text{G}}\;(\text{g}/\text{d})=183.6_{\left(\pm 22.5333\right)}\times \text{EBG}-2.0693_{\left(\pm 4.7254\right)}\times \text{RE.}$$

The k_PG_ was 0.39, and this value was obtained based on the slope of the linear regression between RP and MPI.

The male dairy cattle (Holstein and Holstein crossbreeds) play important participation in feedlots in Brazil and the USA [3–5]. However, although it is acknowledged that dairy and beef cattle exhibit differences regarding their nutritional requirements [1,2], most of nutritionists use some version beef cattle NRC as source of information on nutrient requirements to formulate diets [3,6]. In this context, we compared energy and protein requirements estimated by equations proposed in this study with the values estimated by the last update of beef cattle NRC (NASEM) [1], which have maintained the same recommendations of the previous version [20] for energy and protein requirements.

Considering a steer with 416 kg of SBW, EBW of 365, shrunk body gain of 1.38 kg/d, EBG of 1.29 kg/d (the average of the steers in this study), and equivalent EBW (EQEBW) of 355 kg (EQEBW was calculated considering the BW at maturity of 532 kg and standard reference weight of 517 [2]), the values calculated by our equations and the same values estimated with NASEM [1] equations were: 6.93 and 7.09 Mcal/d for NE_M_, 4.95 and 6.86 Mcal/d for NE_G_, 367 and 350 g/d for MP_M_, and 227 and 168 g/d for NP_G_. In comparison to the values calculated by the equations proposed in this study, the NE_M_ and NE_G_ estimated by NASEM [1] were approximately 0.16 and 1.91 Mcal/d greater, respectively. However, the MP_M_ and NP_G_ estimated by NASEM [1] were approximately 17 and 59 g/d below the value calculated by equations developed in the current study.

The most substantial differences between the requirements estimated by the equations proposed in this study and NASEM [1] are regarding NE_G_ and NP_G_. The composition of EBG is the main determinant for estimating the nutrient requirements for BW gain, and it depends on the physiological maturity of the cattle, which is affected by sex and breed [25]. Differently from our database, the equations recommended by NASEM [1] are derived from studies based on body composition of *Bos taurus* beef purebreds and their crossbreds. Differences in maturity degree of the different genetic groups reflect different body composition in animals with the same BW [2]. Thus, for animals with the same BW and weight gain rate, which is the case of the example above, higher energetic and lower protein concentrations are expected in the gain, and consequently greater energy and lower protein requirements for gain in animals from genetic groups with lower weight at maturity (medium-frame steers; i.e. beef steers), compared with genetic groups with later maturity (large-frame steers; i.e. dairy steers) [2]. Therefore, the differences observed between the nutrient requirements obtained in the current study and those values estimated by NASEM [1] might be due cattle genetics. Nevertheless, it is important to highlight that the dietary management applied to animals in the current study may also have influenced the nutrient requirement estimations.

### Carcass chemical composition

The HH46 [7] equation did not correctly estimate carcass chemical content of CP and EE (Table 4) because it rejected the null hypothesis of a respective intercept and slope equal to 0 and 1 (p<0.05). Comparing the maximum values estimated with the observed values, HH46 [7] equations overestimated CP and EE by 8.60% and 16.6%, respectively. Good precision and accuracy were observed when estimating water carcass content with the HH46 [7] equation, as CCC was close to 1; and it did not reject the null hypothesis of a respective intercept and slope equals to 0 and 1 (p>0.05). However, CS16 [12] equations had problems of adjustment for carcass water content according to the slope of regression analysis (p<0.05), indicating poor accuracy.

The CS16 [12] equations accurately and precisely estimated CP and EE in carcasses, as the null hypothesis of a respective intercept and slope equal to 0 and 1 (p>0.05) were not rejected; and CCC was close to 1 (CCC of 0.91 and 0.91 for CP and EE, respectively). The accuracy of prediction equations tends to decrease when they are used to estimate carcass composition of animals from genetic groups different from the ones used to generate the equation in the first place [26, 27]. The HH46 [7] equations were developed based on carcass chemical composition of *Bos taurus* beef purebreds and their crossbreds. At the same BW, beef carcasses (Angus) have greater soft tissue to bone ratio, and proportionally more fat than dairy carcasses (Holstein) [28]. These differences are likely because genetic selection in dairy cattle has been focused on milk production rather than tissues deposition. Therefore, the presence of the Holstein breed in the genotype of the animals of the current study, may have resulted in a different growth and conformation pattern when compared to breeds selected for beef production and their crossbreds. Similar to our results, Nour and Thonney [29] pointed out that differences among breed types must be considered for greater accuracy and precision of prediction when using HH46 [7] equations to predict carcass composition of Angus and Holstein steers. Nevertheless, it is important to highlight that the dietary management applied to animals in the current study may also have influenced the body composition obtained.

The CS16 [12] equations were developed using a database composed by animals Holstein×Zebu, which probably resulted in the best goodness of fit for CP and EE content in carcass among the equations evaluated. Therefore, CS16 [12] equations are more appropriate to predict CP and EE, whereas HH46 [7] equations better explained the variation in carcass water content of Holstein×Zebu steers.

### Empty body chemical composition

The CS16 [12] equations did not reject the null hypothesis of a respective intercept and slope equal of 0 and 1 (p>0.05), and presented high R^2^ (R^2^ of 0.97, 0.96, and 0.99 for CP, EE, and water, respectively) and CCC (CCC of 0.85, 0.96, and 0.98 for CP, EE, and water, respectively) values for all chemical contents evaluated (Table 4).

Moreover, the prediction errors for EE and water empty body contents were mostly (more than 75% of MSEP) associated with random errors, rather than central tendency, and due to linear regression or systematic bias. However, most of prediction error for CP in the empty body (more than 80% of MSEP) was associated with the SB.

The CS16 [12] equations are part of the suggested equations in the 3rd edition of Nutrient Requirements of Zebu and Crossbred Cattle [2] for estimating body composition of Zebu and crossbred cattle. The CS16 [12] equations were developed after authors verified that equations generated based on a database composed by Zebu cattle (mainly Nellore) and their crosses with *Bos taurus* beef breeds (Angus or Simmental) [30] did not correctly estimate body composition of crossbred dairy cattle. However, until the present study, CS16 [12] equations had not been evaluated with an independent database.

In the current study, CS16 [12] equations accurately estimated all chemical components of empty body. However, reduced goodness of fit was observed when predicting CP content. The CS16 [12] developed equations using body composition data from crossbred dairy cattle, which were composed by 80 bulls, 56 steers, and 44 heifers. The greater proportion of bulls in the database of CS16 [12] could explain the reduced goodness of fit in predicting empty body CP in the current study, as bulls usually have greater CP content and accretion rates than steers [25]. However, when predicting empty body CP sex was not shown to be significant [12].

In summary, the NE_M_ and MP_M_ of Holstein×Zebu steers were 0.083 Mcal/EBW^0.75^/d and 4.40 g/EBW^0.75^, respectively. The NE_G_ and NP_G_ can be estimated with the following equations:

$$\begin{array}{l} {\text{NE}}_{\text{G}}\;(\text{Mcal}/\text{d})=0.2973\times {\text{EBW}}^{0.4336}\;\text{and}\\ {\text{NP}}_{\text{G}}\;(\text{g}/\text{d})=183.6\times \text{EBG}-2.0693\times \text{RE.} \end{array}$$

Moreover, our results suggested that there may be differences between the NE_G_ and NP_G_ of beef and dairy crossbred steers. Thus, the equations described above may be used to calculate the nutrient requirements of Holstein×Zebu steers instead of using recommendations based on values from beef cattle.

The CP and EE chemical contents in carcass, and all chemical components in the empty body were precisely and accurately estimated by CS16 [12] equations. However, water content in carcass was better predicted by HH46 [7] equation. Therefore, the equations proposed by CS16 [12] were the best estimator for CP and EE in carcass of crossbred dairy steers, whereas water in carcass is better estimated using equations proposed by HH46 [7]. The equations proposed by CS16 [12] can be used to estimate all chemical components in the empty body.

## Figures and Tables

**Table 1:** Composition of experimental diet

Item	% Dry matter
Diet composition	
Corn silage	40.0
Ground corn	53.6
Soybean meal	3.60
Urea	0.90
Ammonium sulfate	0.10
Sodium bicarbonate	0.75
Magnesium oxide	0.25
Mineral mixture1)	0.13
Salt	0.30
Limestone	0.42
Analyzed composition	
Dry matter	43.5
Organic matter	94.2
Crude protein	11.0
Ether extract	2.95
Neutral detergent fiber2)	27.6
Non-fiber carbohydrates	54.3
Energy density (Mcal/kg DM)	
NE_M_	1.91
NE_G_	1.05

1) Composition: 0.03% Na as Na_2_SeO_3_; 93.7% S as CuSO_4_; ZnSO_4_, MnSO_4_ and CoSO_4_; 0.48% Cu as CuSO_4_; 4.71% Zn as ZnSO_4_; 0.77% Mn as MnSO_4_; 0.06% Se as Na_2_SeO_3_; 0.25% Co as CoSO_4_.

2) Corrected to ash and protein.

**Table 2:** Variable description used to estimate carcass and empty body chemical composition of Holstein×Zebu steers (n = 33)

Item	Mean	SD	Maximum	Minimum
Empty body weight (kg)	365	67.1	530	196
Organs and viscera (% EBW)	18.0	2.08	22.0	12.6
Visceral fat (% EBW)	6.28	2.20	9.81	0.72
9th to 11th rib section crude protein (%)	16.7	1.88	19.2	11.4
9th to 11th rib section ether extract (%)	22.4	7.34	35.8	6.72
9th to 11th rib section water (%)	56.7	6.91	77.3	42.62

EBrW, empty body weight.

**Table 3:** Equations used to estimate carcass and empty body chemical composition of Holstein × Zebu steers

Item	Equations
Carcass chemical composition	
Hankins and Howe [7]	CP_carc_ = 5.98+0.66×CP_RS_
	EE_carc_ = 2.82+0.77×EE_RS_
	W_carc_ = 14.90+0.78×W_RS_
Costa e Silva et al [12]	CP_carc_ = 18.38+0.16×CP_RS_−0.20×OV
	EE_carc_ = 4.54+0.48×EE_RS_+0.12×OV
	W_carc_ = 55.67−0.21×W_RS_−0.021×EBW
Empty body chemical composition	
Costa e Silva et al [12]	CP_EBW_ = 19.92+0.086×CP_RS_−0.19×OV
	EE_EBW_ = 3.53+0.34×EE_RS_+0.80×VF+0.10×OV
	W_EBW_ = 53.02+0.17×W_RS_−1.28×VF+0.27×OV

CP_carc_, crude protein in the carcass (%); CP_RS_, crude protein in the 9th–11th rib section (%); EE_carc_, ether extract in the carcass (%); EE_RS_, ether extract in the 9th–11th rib section (%); W_carc_, water in the carcass (%); W_RS_, water in the 9th–11th rib section (%); EBW, empty body weight (kg); OV, percentage of organs and viscera in the EBW (% EBW); VF, percentage of mesenteric fat plus renal, pelvic, and cardiac fat in the EBW (% EBW); CP_NC_, crude protein in the non-carcass (kg); EE_NC_, ether extract in the non-carcass (kg); W_NC_, water in the carcass (kg); CP_EBW_, crude protein in the empty body (%); EE_EBW_, ether extract in the empty body (%); and W_EBW_, water in the empty body (%).

**Table 4:** Mean (kg) and descriptive statistics for relationship among the observed and predicted values to carcass and empty body chemical composition

Item	Carcass									Empty body					
	
	Crude protein			Ether extract			Water			Crude protein		Ether extract		Water	
					
	Obs.1)	HH462)	CS162)	Obs.1)	HH462)	CS162)	Obs.1)	HH462)	CS162)	Obs.1)	CS162)	Obs.1)	CS162)	Obs.1)	CS162)
Mean	35.4	36.3	37	39.8	44.6	38.5	130	125.1	127	58.6	64	69.4	67.3	215	213
SD	5.68	6.96	6.04	11.7	16.2	12.1	20.3	18.8	18.3	10.3	10.3	22.7	23.1	31.1	29.7
Maximum	43.5	47.6	44.9	62.6	73.8	59.8	155	159	152	74.1	78.8	112	111	258	256
Minimum	22.7	18.1	21.7	14.1	11.3	13.1	72	74.7	77.6	34.9	37.4	22.8	19.4	122	129
R^2^	-	0.9	0.94	-	0.88	0.91	-	0.92	0.98	-	0.97	-	0.96	-	0.99
CCC	-	0.88	0.91	-	0.79	0.91	-	0.89	0.97	-	0.85	-	0.96	-	0.98
Regression															
Intercept															
Estimate	-	8.59	2.49	-	11.4	5.57	-	5.44	−9.23	-	−3.33	-	5.8	-	−4.76
Standard error	-	2.386	2.151	-	2.92	2.895	-	10.088	5.263	-	2.93	-	3.479	-	6.147
p-value3)	-	0.001	0.257		<0.001	0.064	-	0.593	0.09	-	0.265	-	0.106	-	0.445
Slope															
Estimate	-	0.74	0.89	-	0.64	0.89	-	0.99	1.09	-	0.97	-	0.94	-	1.03
Standard error	-	0.065	0.057	-	0.062	0.072	-	0.08	0.04	-	0.045	-	0.049	-	0.029
p-value4)	-	<0.001	0.06	-	<0.001	0.127	-	0.923	0.036	-	0.484	-	0.264	-	0.248
MSEP	-	9.73	6.59	-	86.5	25.9	-	85.2	23.8	-	35.3	-	44.4	-	27.8
SB	-	0.84	2.77	-	22.8	1.53	-	20	4.9	-	28.9	-	4.2	-	5.85
MaF	-	3.22	0.44	-	33.6	1.79	-	0.02	2.61	-	0.1	-	1.61	-	0.97
MoF	-	5.68	3.37	-	30.1	22.6	-	65.2	16.3	-	6.26	-	38.6	-	21

SD, standard deviation; CCC, correlation and concordance coefficient; MSEP, mean square error of prediction; SB, squared bias; MaF, magnitude of random fluctuation; MoF, model of random fluctuation.

1) Obs, observed values.

2) HH46, equations proposed by Hankins and Howe [7]; CS16, equations proposed by Costa e Silva et al [12].

3) Ho:β0 = 0.

4) Ho:β1 = 1.
